# Extensive perinephric hematoma following excessive irrigation pressure during flexible ureteroscopy: case report of a preventable complication

**DOI:** 10.1186/s13037-026-00479-x

**Published:** 2026-03-07

**Authors:** Chinnakhet Ketsuwan

**Affiliations:** Division of Urology, Department of Surgery, Faculty of Medicine Ramathibodi Hospital, Mahidol University, 270 Rama VI Road, Toong Phayathai, Ratchathewi, Bangkok, 10400 Thailand

**Keywords:** Flexible ureteroscopy, Intrarenal pressure, Perinephric hematoma, Patient safety, Root cause analysis

## Abstract

**Background:**

Flexible ureteroscopy (fURS) is widely regarded as a safe and effective procedure for the management of urolithiasis. Nevertheless, inadequate management of intrarenal pressure during fURS represents an underrecognized patient safety hazard. The use of high-pressure irrigation to compensate for poor visualization—particularly when outflow is restricted—may expose patients to prolonged supraphysiologic intrarenal pressure and preventable harm. I report a severe pressure-related renal injury following fURS and analyze the event using a human factors and systems-based root cause analysis framework.

**Case presentation:**

A 45-year-old man underwent fURS for a 1.5-cm proximal ureteral stone. A ureteral access sheath was not used due to a tight distal ureter. During the 100-min procedure, visualization progressively deteriorated because of mucosal edema, bleeding, and debris. Irrigation was escalated using a manual pressure bag and reported intraoperatively as very high (300–400 mmHg on the pressure bag gauge) for a prolonged period. Shortly following surgery, the patient developed severe flank pain and a high-grade fever. Computed tomography revealed a massive perinephric hematoma without active contrast extravasation. The patient remained hemodynamically stable and improved with conservative management.

**Conclusion:**

The described event is best understood as a preventable iatrogenic injury arising from a predictable hazard: pressure escalation to restore visualization. It occurred in a system without adequate defenses, namely, limited outflow, a lack of real-time pressure feedback, the absence of pressure or time stop rules, and insufficient team cross-checks against cognitive fixation. Intrarenal pressure should be treated as a critical safety variable in endourology. Pressure-governed workflows—prioritizing outflow augmentation, objective monitoring when feasible, and escalation pathways that favor staging over unmonitored pressure escalation—are essential to prevent similar harm.

## Background

Flexible ureteroscopy (fURS), which has been enabled by advances in endoscope design, laser lithotripsy, and ancillary devices, has become a cornerstone procedure for the management of upper urinary tract calculi [1, 2]. Although major complications are uncommon, fURS inherently depends on continuous irrigation during the procedure to maintain visualization. This creates a latent safety hazard: intrarenal pressure may rise far beyond physiologic levels when inflow exceeds outflow.

Physiologic renal pelvic pressure is typically low, often cited as < 10 mmHg [3]. Elevations beyond 30–40 mmHg are associated with pyelorenal and pyelovenous reflux, which facilitate the systemic translocation of bacteria and endotoxins and increase the risk of infection [4–6]. Under conditions of restricted outflow, such as ureteral edema, instrument obstruction, or the absence of a ureteral access sheath, high external irrigation pressure may translate into markedly elevated intrarenal pressure [3, 4]. While infection is the most recognized pressure-related complication, sustained supraphysiologic intrarenal pressure may also contribute to renal parenchymal injury and hemorrhagic events.

In routine clinical practice, irrigation pressure is frequently not measured, particularly when gravity systems or manual pressure bags are used [7]. As a result, irrigation pressure to restore visualization may escalate without objective feedback or defined limits. This case report reframes a massive perinephric hematoma following fURS not as an unavoidable rarity, but as a preventable iatrogenic injury enabled by modifiable system vulnerabilities in irrigation pressure management.

## Case presentation

**Fig. 1:**
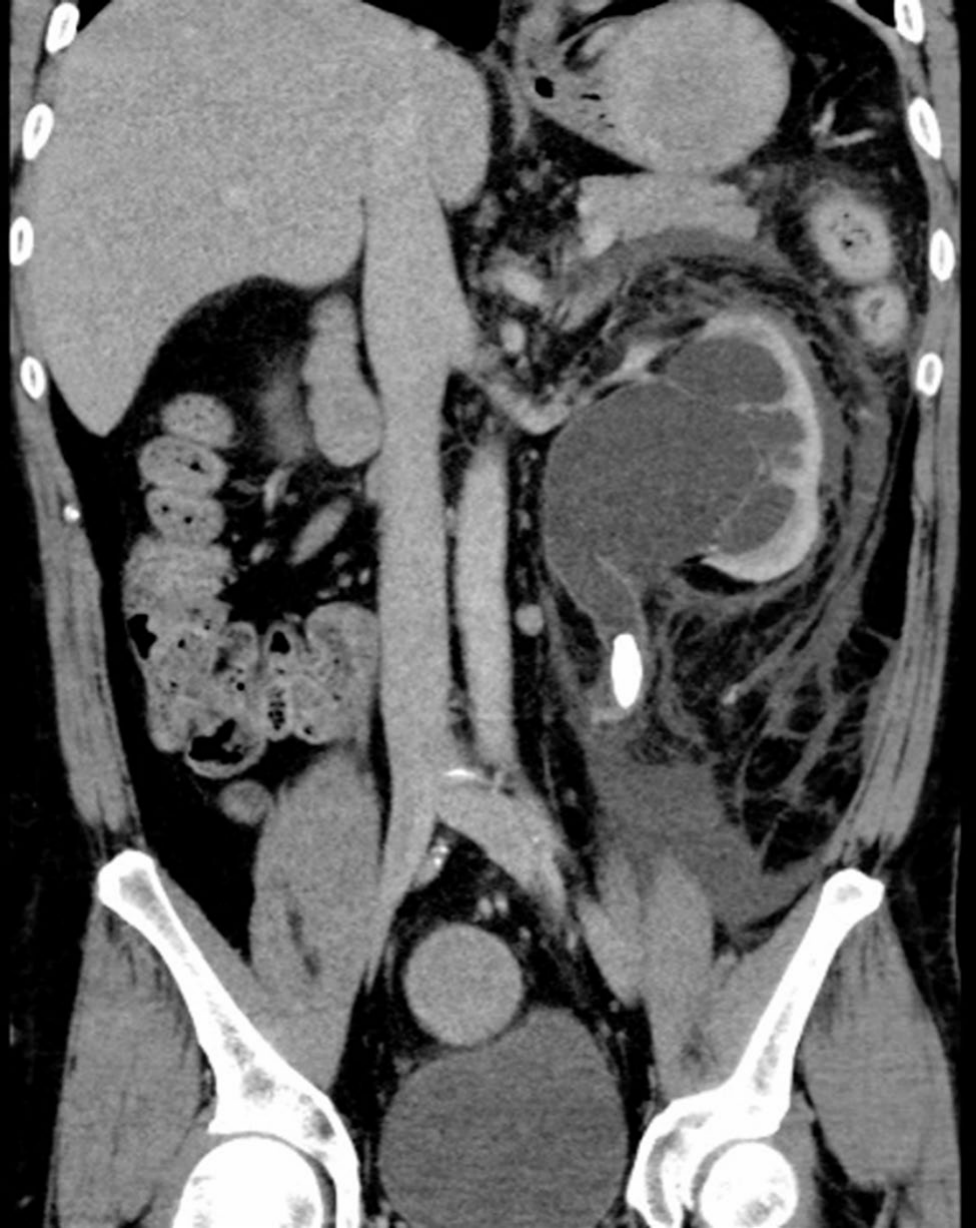
Preoperative computed tomography showing a proximal left ureteral calculus with moderate hydronephrosis

A 45-year-old man with no history of bleeding diathesis presented with left flank pain. Preoperative non-contrast computed tomography (CT) revealed a 1.5-cm proximal left ureteral stone with moderate hydroureteronephrosis (Fig. 1). No perinephric fluid collection or hematoma was present. The patient’s baseline hemoglobin was 14.5 g/dL, with a normal platelet count and renal function and coagulation parameters. The urinalysis and urine culture were negative.

The patient underwent fURS via holmium laser lithotripsy using a 7.5-Fr digital flexible ureteroscope. A ureteral access sheath was not placed because of resistance caused by a tight distal ureter. During the procedure, the stone migrated into the renal pelvis. Visualization progressively deteriorated owing to mucosal edema, bleeding, and floating debris. To maintain endoscopic visibility, irrigation was escalated using a manual pressure bag. The intraoperative team reported the bag gauge reading to be very high (300–400 mmHg) for a prolonged portion of the 100-min procedure. No ureteral perforation or direct laser injury was identified. A double-J ureteral stent was placed at the conclusion of the procedure.

**Fig. 2:**
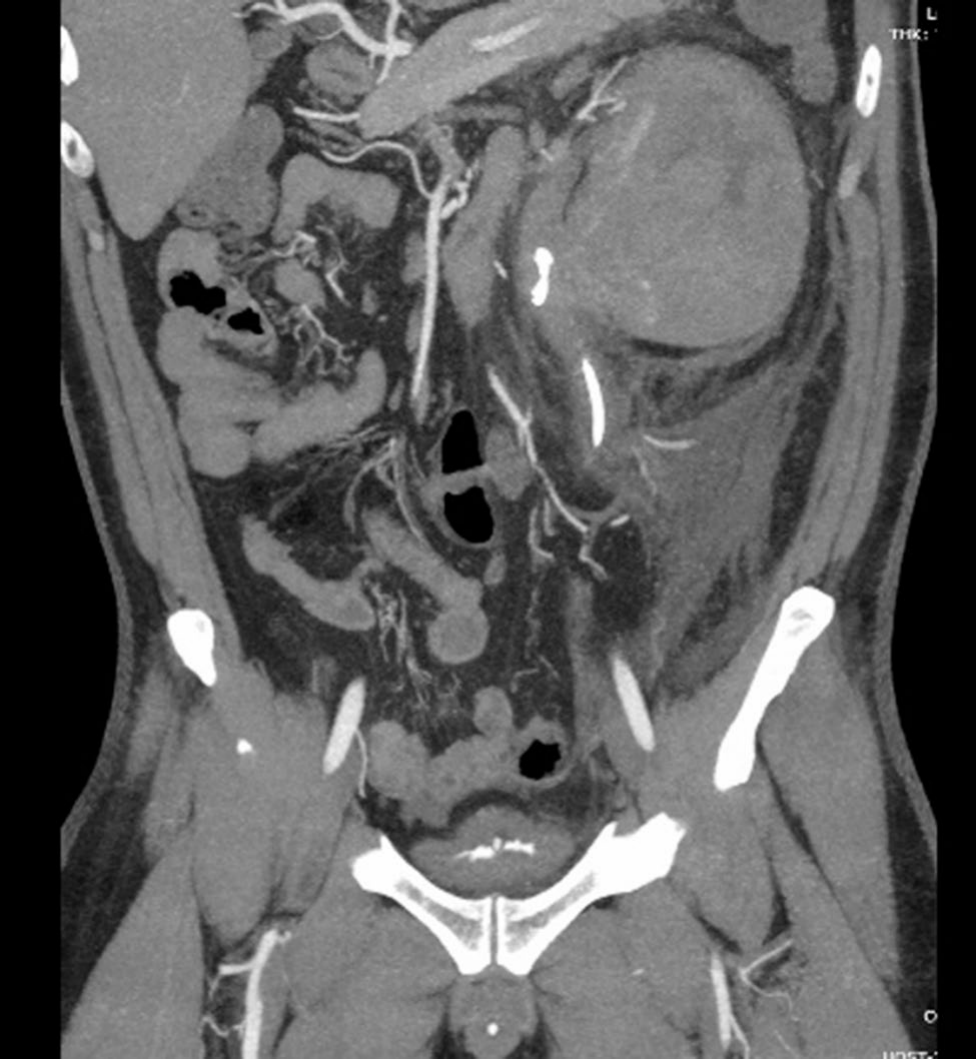
Postoperative computed tomography demonstrating a massive perinephric hematoma

Postoperatively, the patient developed severe flank pain and high-grade fever. CT imaging revealed a massive left perinephric hematoma compressing the renal parenchyma, without evidence of active contrast extravasation (Fig. 2). He remained hemodynamically stable. Conservative management consisted of bed rest, serial hemoglobin monitoring, and the administration of analgesics and intravenous antibiotics. The clinical course improved gradually, and no transfusion, angioembolization, or surgical intervention was required. The patient was discharged on postoperative day 10, and the ureteral stent was removed uneventfully two months later.

### Definitions and root cause analysis

#### Event definition

Index harm Massive perinephric hematoma following fURS, temporally associated with prolonged high-pressure irrigation in the setting of restricted outflow and the absence of real-time pressure feedback.

#### Why this constitutes a patient safety event

Perinephric hematoma following ureteroscopy is rare. However, the exposure observed in this case, namely, prolonged high-pressure irrigation with impaired outflow, represents a known and modifiable hazard. The adverse outcome occurred when multiple defenses against pressure-related harm were absent or failed simultaneously, which is consistent with established systems accident models.

### Timeline and safety-relevant decision points

- Preoperative: Low baseline bleeding risk, negative urine culture, moderate hydronephrosis present.
- Intraoperative (early): Ureteral access sheath not placed due to tight distal ureter → outflow protection weakened.
- Intraoperative (mid): Visualization deteriorated (bleeding, debris, edema) → hazard signal.
- Intraoperative (escalation): Manual pressure bag used at very high settings for a prolonged period → unmonitored exposure.
- Postoperative: Flank pain and fever → CT confirmed massive perinephric hematoma → conservative management.

The critical pivot occurred when deteriorating visualization prompted the escalation of irrigation pressure rather than a structured safety pathway that emphasized outflow augmentation or staging.

### Contributing factors (Systems Engineering Initiative for Patient Safety framework)

#### Tools/technology

- Manual pressure bag capable of delivering high inflow without closed-loop safety limits.
- No objective or real-time monitoring of intrarenal pressure.
- Restricted outflow without ureteral access sheath or suction assistance.

#### Team/communication

- No formal verbal call-outs when initiating or sustaining high-pressure irrigation.
- Lack of structured team cross-checks to challenge the continued escalation.

#### Human factors

- Task fixation and plan continuation bias, with a focus on restoring visualization and completing the lithotripsy.

#### Organization

- Absence of institutional guidance or protocols addressing irrigation pressure limits and documentation.

### Barrier analysis

**Fig. 3:**
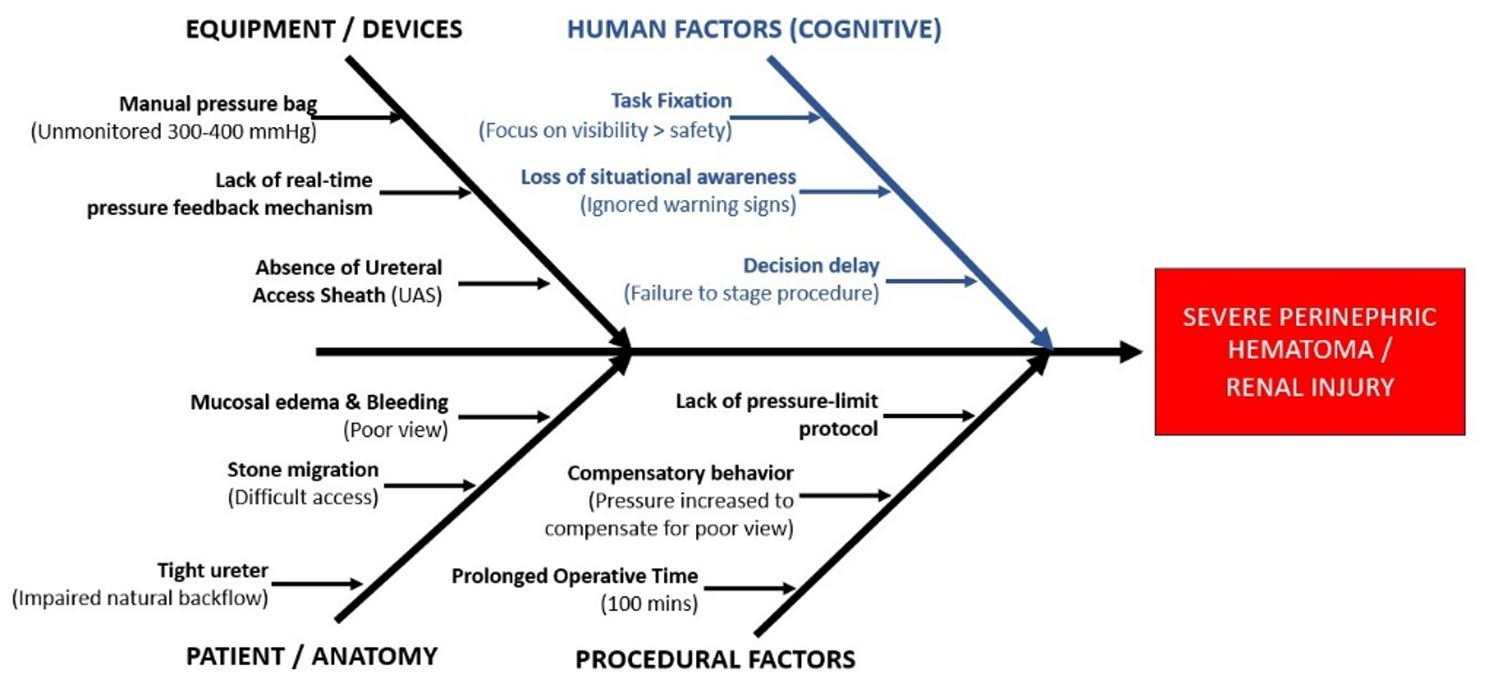
Swiss-cheese root cause analysis of a preventable irrigation pressure-related renal injury during flexible ureteroscopy

The described event can be conceptualized as an alignment of failures across multiple defensive layers—outflow protection, monitoring, protocolized limits, and team cross-checks—which permitted prolonged high-pressure irrigation exposure and subsequent renal/perirenal hemorrhagic injury (Fig. 3). A detailed root cause analysis matrix is presented in Table 1.

**Table 1:** Root cause analysis matrix

Domain	Contributing factor	Evidence in this case	Risk mechanism	Corrective and preventive action
Patient/anatomy	Narrow distal ureter	Not possible to place ureteral access sheath initially	Restricted outflow increases intrarenal pressure for any given irrigation inflow	Reassess ureteral access sheath placement after dilation; consider a smaller ureteral access sheath, staged procedure, or suction-based alternatives when a ureteral access sheath cannot be placed
	Hydronephrosis	Moderate hydronephrosis on preoperative computed tomography (CT)	Larger collecting system volume leads to higher pressure accumulation during sustained irrigation	Preoperative risk stratification for susceptibility to high pressure; plan outflow strategy in advance
Task/process	Poor visualization (bleeding, debris, edema)	Worsening endoscopic view prompted irrigation pressure escalation	Drives compensatory behavior (irrigation pressure escalation) that increases intrarenal pressure	Implement a “poor-visibility pathway”: pause, reduce irrigation pressure, clear debris, reassess bleeding, improve outflow, or stage
	Prolonged operative time	100-min procedure	Longer exposure increases cumulative pressure-related tissue injury	Introduce pressure/time stop rules that require reassessment or staging after predefined thresholds
Tools/technology	Manual pressure bag with no safety limits	Bag gauge reported at 300–400 mmHg for a prolonged period	High, unregulated inflow can generate supraphysiologic intrarenal pressure when outflow limited	Prefer closed-loop pumps with alarms; restrict manual pressure bags to short, low-pressure use with supervision
	No real-time intrarenal pressure feedback	No intrarenal pressure monitoring or alarms	Sustained dangerous intrarenal pressure goes undetected	Introduce inline manometer or pressure-sensing devices where feasible; document pressure strategy
	No facilitated outflow	No ureteral access sheath; no suction used	Inflow that exceeds egress leads to rising intrarenal pressure	Use ureteral access sheath, suction-enabled sheaths, or in-scope suction when debris/bleeding compromises view
Team/communication	No high pressure irrigation call-outs	No documented team acknowledgment of prolonged high-pressure irrigation	Team lacks shared awareness of a safety threshold being crossed	Standardize verbal “high-pressure irrigation” call-outs and timed reassessments (e.g., every 10–15 min)
	No challenge response	No evidence of team challenge to continued pressure escalation	Cognitive fixation remains unchecked	Implement two-challenge rule or safety stop when high-risk conditions persist
Human factors	Task fixation	Focus on restoring visibility and completing the lithotripsy	Plan continuation bias delays safer alternatives (staging, outflow augmentation)	Train teams on cognitive traps; embed stop rules in protocols rather than relying on individual vigilance
	Loss of situational awareness	Irrigation pressure treated as a tool rather than a hazard	Intrarenal pressure not conceptualized as a “vital sign”	Reframe intrarenal pressure as a monitored safety variable in ureteroscopy checklists
Organization	No protocol for irrigation pressure management	No documented limits for irrigation pressure or duration	Hazard is normalized and unmanaged	Create institutional standards for irrigation devices, pressure limits, documentation, and escalation ladders
	No documentation of the irrigation strategy	Irrigation method/pressure not formally recorded	Limits learning and quality improvement	Require documenting of irrigation mode, ureteral access sheath use, and outflow plan in operative notes

### Tasks/processes

- Absence of an explicit escalation ladder or stop rules for irrigation pressure and exposure times.

## Discussion

Although uncommon across the various nephrolithiasis treatment modalities, perinephric and subcapsular renal hematomas have been reported [8–11]. These events have been described in flexible ureteroscopy, particularly in association with prolonged operative times, high irrigation pressures, and impaired outflow [12]. This case adds a patient safety perspective by demonstrating how a predictable hazard—irrigation pressure escalation to restore visualization—can lead to severe harm when the system defenses are inadequate.

A critical analytic distinction lies between external irrigation pressure and true intrarenal pressure. Although the gauge of a pressure bag does not directly equate to intrarenal pressure, experimental and clinical data indicate that under conditions of restricted outflow, intrarenal pressure may rise substantially with high external irrigation pressure and prolonged exposure [3, 4]. In this case, several risk factors converged: the absence of a ureteral access sheath, mucosal edema and bleeding, the debris burden, and a prolonged procedure. Together, these factors plausibly resulted in sustained supraphysiologic intrarenal pressure that was sufficient to contribute to forniceal and vascular injury consistent with the observed perinephric hematoma.

From a human factors perspective, task fixation offers a parsimonious explanation for the persistent irrigation pressure escalation in this case. As visualization worsened, the operational goal may have narrowed to “restore view and complete [the] lithotripsy,” which would have delayed safer alternatives, such as staging the procedure, reassessing the ureteral access sheath placement, or employing suction-assisted techniques [2, 6, 13]. Importantly, framing this event solely as an operator error would miss the dominant safety lesson: the system allowed high-risk irrigation pressure exposure to continue because objective feedback, protocols, and cross-checks were absent—a classic pattern in preventable adverse events [14].

The implications of these findings extend beyond this single case. Irrigation practices during fURS vary widely, and unmonitored pressure escalation is common in real-world settings [7]. A pragmatic prevention strategy could be to apply an ALARA-like principle to intrarenal pressure, namely, to minimize irrigation pressure, maximize outflow, and default to staging rather than prolonged unmonitored escalation. When feasible, ureteral access sheath placement reduces intrarenal pressure and improves outflow. However, when a ureteral access sheath cannot be placed, alternative strategies, such as intermittent decompression, suction-enabled devices, and staged management, should be considered [4, 15–17]. This analysis was constrained by the absence of direct intrarenal pressure measurements and reliance on reported readings from the pressure bag gauge. While causality cannot be proven definitively, the temporal relationship, biologically plausible mechanism, and convergence of known risk factors support a systems-based inference that the irrigation pressure-related injury was likely contributory and preventable.

## Conclusions

This case demonstrates that excessive irrigation pressure during fURS represents an underrecognized yet preventable patient safety hazard. Poor visualization should trigger a structured safety response rather than the escalation of irrigation pressure as the default response. Recognizing intrarenal pressure as a critical safety variable—and implementing pressure-governed workflows with outflow optimization, objective feedback when feasible, protocolized limits, and team cross-checks—may reduce the risk of iatrogenic renal injury.

## Data Availability

The datasets used during the current study are available from the corresponding author on reasonable request.

## Abbreviations

ALARA: As Low As Reasonably Achievable
CT: Computed Tomography
fURS: Flexible Ureteroscopy
